# Association between hemoglobin-to-red blood cell distribution width ratio and rheumatoid arthritis in US adults: evidence from the national health and nutrition examination survey 2009–2018

**DOI:** 10.1016/j.pmedr.2025.103169

**Published:** 2025-07-09

**Authors:** Nian Kuang, Jing Liu, Zhaoduan Hu, Yanxia Wu, Rui Peng

**Affiliations:** a College of Acupuncture-Moxibustion and Orthopedics, Hubei University of Chinese Medicine, Wuhan 430065, China; b Department of General Practice, Traditional Chinese and Western Medicine Hospital affiliated to Hubei University of Chinese Medicine, Wuhan 430022, China

**Keywords:** Rheumatoid arthritis, Hemoglobin-to-red blood cell distribution width ratio, Epidemiology, NHANES, Cross-sectional study

## Abstract

**Objective:**

This cross-sectional study aimed to investigate the association between the hemoglobin-to-red blood cell distribution width ratio (HRR) and rheumatoid arthritis in adults.

**Methods:**

Cross-sectional data from 22,352 participants in the National Health and Nutrition Examination Survey (NHANES) 2009–2018 were analyzed. HRR was defined as hemoglobin concentration (g/dL) divided by red blood cell distribution width (%) and grouped into quartiles. The multivariable logistic regression model and restricted cubic spline (RCS) models assessed the connection between HRR and rheumatoid arthritis, adjusted for demographics, socioeconomic factors, and comorbidities.

**Results:**

Our findings reveal a significant negative correlation between HRR measurements and rheumatoid arthritis susceptibility. Higher HRR quartiles showed progressively lower rheumatoid arthritis risk (quartile 4 vs. quartile 1: OR = 0.68, 95 % CI: 0.56,0.83). RCS revealed an inverse linear association after adjustment (P for overall <0.001; P for nonlinear = 0.174).

**Conclusions:**

HRR is inversely associated with rheumatoid arthritis risk, suggesting its potential as a biomarker for rheumatoid arthritis risk stratification. Nevertheless, additional investigations are required to corroborate these observations.

## Highlights

• Hemoglobin-to-red cell distribution width ratio (HRR) may be a potential biomarker for rheumatoid arthritis risk assessment.
• Multivariable logistic regression showed a significant inverse association between HRR and rheumatoid arthritis risk.
• Restricted cubic spline analysis confirmed a linear inverse relationship between HRR and rheumatoid arthritis risk.

## 1 Introduction

Rheumatoid arthritis is a systemic autoimmune disorder characterized by chronic joint inflammation and extra-articular manifestations (Di Matteo et al., 2023). With a global prevalence ranging from 0.25 % to 1 %, this condition exhibits significant gender disparity, demonstrating a 2–3 fold higher occurrence in women than in men (Di Matteo et al., 2023; Black et al., 2023). Left untreated, rheumatoid arthritis progresses to irreversible joint damage and structural deformities, culminating in permanent disability, refractory pain syndromes, and increased mortality risk (Black et al., 2023). Pathophysiological mechanisms involve dysregulation of T lymphocyte subsets, particularly T helper 17/regulatory T cell imbalance, coupled with sustained production of pro-inflammatory cytokines including interleukin-6 (IL-6) and tumor necrosis factor-α (TNF-α), which collectively mediate synovial destruction (Hu et al., 2024; Paradowska-Gorycka et al., 2020; Kishimoto and Kang, 2022). The chronic inflammatory state further induces anemia of chronic disease (ACD) through dual mechanisms: suppression of erythropoietin bioactivity and hepcidin-mediated sequestration of iron within macrophage stores, thereby creating functional iron deficiency in erythroid progenitor cells (Papadaki et al., 2002).

As a cornerstone parameter for anemia assessment, decreased hemoglobin levels not only reflect the severity of anemia of chronic disease but also demonstrate strong associations with hypoxia-driven inflammatory amplification (Lanser et al., 2021; De Souza et al., 2022). Red blood cell distribution width (RDW) quantifies erythrocyte volume heterogeneity and has served as a sensitive diagnostic parameter for evaluating systemic inflammation and oxidative damage (Liu et al., 2021). Clinical evidence indicates that elevated RDW serves as an independent prognostic marker in acute myeloid leukemia and severe sepsis, while reduced hemoglobin levels correlate with increased long-term mortality risk in chronic obstructive pulmonary disease, where even mild anemia confers significant clinical risk (Kim et al., 2013; Vucinic et al., 2021; Park et al., 2018). Recent studies highlight the superior prognostic value of the hemoglobin-to-red blood cell distribution width ratio (HRR) over individual hematological parameters in cardiovascular, cerebrovascular, and small cell lung carcinoma management, attributable to its unique integration of anemia-related and inflammatory parameters (Wang et al., 2025; Xiong et al., 2024; Wu et al., 2020). This dual-parameter index has consequently been proposed as a novel biomarker for chronic inflammatory disorders. Nevertheless, no systematic investigation has explored the association between HRR and rheumatoid arthritis. This population-based investigation employed a cross-sectional design to examine potential associations between HRR and rheumatoid arthritis using nationally representative data collected through the National Health and Nutrition Examination Survey (NHANES) from 2009 to 2018.

## 2 Methods

### 2.1 Data source and study population

As a large-scale epidemiological survey program, NHANES is implemented by the National Center for Health Statistics (NCHS), a division of the Centers for Disease Control and Prevention (CDC), to systematically assess the health and nutritional status of the U.S. population. Employing a sampling framework combining probability-based selection, stratification, and multiple implementation phases, NHANES annually collects representative health and nutritional data from approximately 5000 civilians, encompassing multidimensional information including demographic characteristics, socioeconomic status, dietary patterns, health questionnaires, physical examinations, and laboratory measurements. The survey's distinctive strength lies in its integration of interview-based data with standardized physical examinations, making it an invaluable resource for assessing disease prevalence, analyzing risk factors, and informing public health policy development. This investigation obtained ethical clearance from the NCHS Research Ethics Review Board, and written informed consent was secured from all enrolled individuals. Complete methodological protocols and anonymized datasets from the NHANES initiative are available for public access through the designated portal: https://wwwn.cdc.gov/nchs/nhanes/default.aspx. This study utilized data from five consecutive cycles of the NHANES from 2009 to 2018 to examine the association between the HRR and rheumatoid arthritis. NHANES data collected after 2018 were excluded due to disruptions caused by the COVID-19 pandemic, particularly inconsistencies in laboratory testing and questionnaire administration, which may have compromised data quality and comparability. HRR is an emerging biomarker that reflects both inflammation and anemia, yet large-scale population-based studies investigating its association with rheumatoid arthritis remain limited. Therefore, analyzing high-quality, methodologically consistent data from multiple pre-pandemic cycles enhances statistical power and strengthens the robustness and interpretability of the findings.

**Fig. 1:**
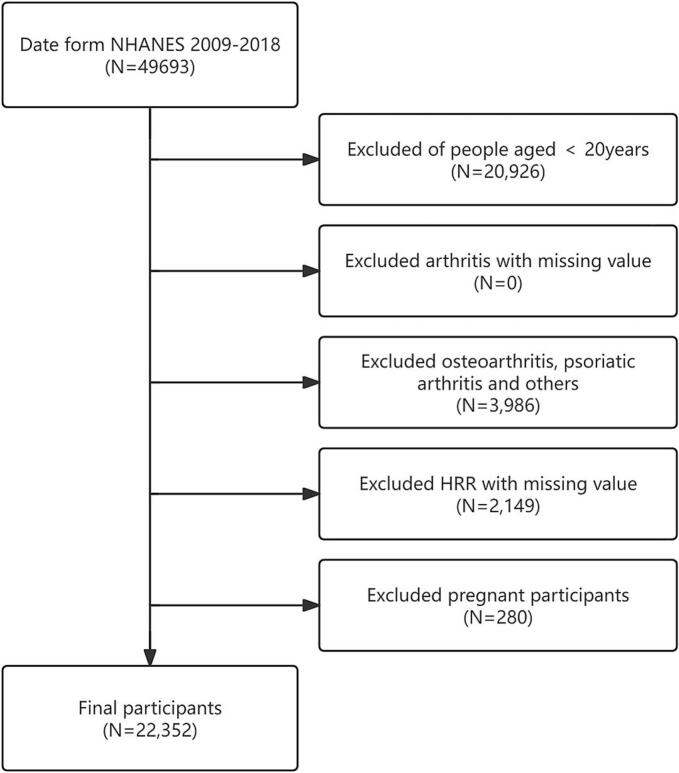
Flowchart of participant selection based on the National Health and Nutrition Examination Survey, 2009–2018, for adults in the United States. Abbreviation: NHANES, National Health and Nutrition Examination Survey; HRR, hemoglobin-to-red blood cell distribution width ratio. (For interpretation of the references to colour in this figure legend, the reader is referred to the web version of this article.)

A standardized set of exclusion criteria was consistently enforced throughout the selection process: (Di Matteo et al., 2023) participants aged <20 years; (Black et al., 2023) individuals with missing arthritis diagnostic data; (Hu et al., 2024) those diagnosed with arthritis types other than rheumatoid arthritis; (Paradowska-Gorycka et al., 2020) cases with missing hemoglobin or red cell distribution width measurements; and (Kishimoto and Kang, 2022) pregnant individuals (Fig. 1). From an initial pool of 49,693 participants, we sequentially excluded 20,926 individuals aged <20 years, 0 cases with missing arthritis data, 3986 individuals with non-rheumatoid arthritis diagnoses, 2149 participants lacking complete hemoglobin and RDW measurements, and 280 pregnant individuals. The final analytical cohort comprised 22,352 eligible participants meeting all inclusion criteria.

### 2.2 Assessment of the HRR and rheumatoid arthritis

The HRR was defined as hemoglobin concentration (g/dL) divided by red blood cell distribution width (%), with these values obtained from the laboratory data (LBXHGB and LBXRDW). Meanwhile, HRR was categorized into quartiles to help identify potential threshold effects in its association with rheumatoid arthritis. This categorization also enhances the robustness of the model by reducing the influence of outliers on the analytical results.

Arthritis diagnosis was ascertained through the self-reported questionnaire (MCQ160a). Specifically, participants were asked, “Has a doctor or other health professional ever told you that you have arthritis?” with response options of “Yes” or “No.” Rheumatoid arthritis status was further determined by the follow-up question: “Which type of arthritis was it?” with response options including “Rheumatoid arthritis,” “Osteoarthritis,” “Psoriatic arthritis,” “Other,” “Refused,” and “Don't know.” This diagnostic approach is supported by previous research demonstrating strong concordance between self-reported arthritis diagnoses and clinically confirmed cases.

### 2.3 Covariates

Demographic and socioeconomic characteristics—including age (20–40, 40–65, and ≥65 years), gender, race, educational attainment, and the family poverty income ratio (PIR)—were obtained from the NHANES demographic data files. PIR was calculated as total household income relative to poverty guidelines, served to stratify the cohort into three socioeconomic tiers: low-income (<1.3), middle-income (1.3–3.5), and high-income (≥3.5) groups.

Lifestyle habits included two variables: smoking status and alcohol consumption. Smoking status was determined based on responses to the question, “Do you now smoke cigarettes?” Alcohol consumption over the previous 12 months was categorized into three groups based on average daily intake: non-drinkers, those consuming 1–3 drinks per day, and those consuming ≥4 drinks per day.

Physical measurements included body mass index (BMI), calculated as weight in kilograms divided by height in meters squared (kg/m^2^). Participants were classified as underweight (<18.5 kg/m^2^), normal weight (≥18.5 and < 25 kg/m^2^), overweight (≥25 and < 30 kg/m^2^), or obese (≥30 kg/m^2^). Additional comorbid conditions, including diabetes and hypertension, were identified based on medical diagnosis.

### 2.4 Statistical analyses

In this study, continuous variables were expressed as Mean ± standard deviation (SD), while categorical variables were presented as frequencies or percentages. Intergroup differences were assessed using chi-square tests and *t*-tests.

Participants were stratified into quartiles based on their HRR values. Quartile 1, with HRR ranging from 0.199 to 0.968, was designated as the reference group. The subsequent quartiles were defined as follows: quartile 2 (0.969 < HRR ≤ 1.07), quartile 3 (1.071 < HRR ≤ 1.17), and quartile 4 (1.171 < HRR ≤ 1.61). To assess the potential independent association between HRR and rheumatoid arthritis, multivariable logistic regression analysis was conducted using two models: Model 1 was unadjusted, while Model 2 was fully adjusted for all covariates. To investigate potential complex nonlinear relationships between HRR and rheumatoid arthritis risk, we employed restricted cubic splines (RCS). Statistical computations were executed using R software (v4.4.2), with results achieving *p*-values below 0.05 deemed statistically significant.

## 3 Results

### 3.1 Baseline characteristics of participants

**Fig. 2:**
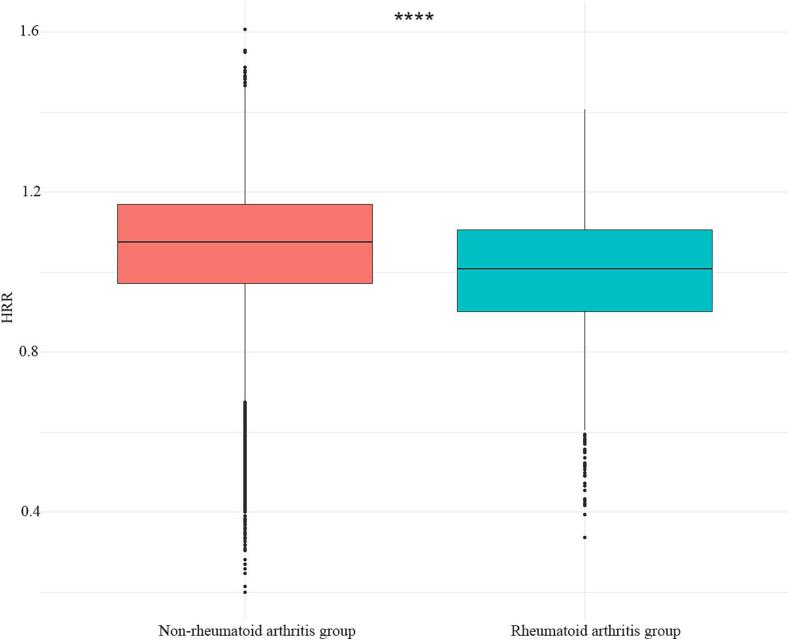
Comparison of hemoglobin-to-red blood cell distribution width ratio between adults with and without rheumatoid arthritis in the United States, based on data from the National Health and Nutrition Examination Survey, 2009–2018. Abbreviation: HRR, hemoglobin-to-red blood cell distribution width ratio; ****: *P* < 0.0001. (For interpretation of the references to colour in this figure legend, the reader is referred to the web version of this article.)

The study cohort comprised 22,352 participants, with a nearly equal gender distribution (50.6 % male and 49.4 % female). Rheumatoid arthritis was diagnosed in 1336 cases, representing a prevalence of 5.98 %. Table 1 presents the clinical characteristics of participants stratified by HRR quartiles, revealing a significant inverse association between HRR quartiles and rheumatoid arthritis prevalence (*P* < 0.001). A progressive decrease in rheumatoid arthritis prevalence was observed with increasing HRR quartiles: quartile 1 (9.39 %) > quartile 2 (6.57 %) > quartile 3 (4.57 %) > quartile 4 (3.36 %), suggesting that individuals with higher HRR values may have a reduced risk of rheumatoid arthritis development. Significant differences in HRR quartiles were observed across various demographic and clinical parameters (all *P* < 0.001), including age, gender, race, poverty-to-income ratio, smoking status, alcohol consumption, body mass index, diabetes status, hypertension status, and educational attainment. Participants in the lower HRR quartiles had a higher propensity to be older, female, non-Hispanic Black, and of lower socioeconomic status. Additionally, this group demonstrated higher rates of obesity, lower educational attainment, and increased prevalence of diabetes and hypertension compared to those in higher HRR quartiles. Additionally, Fig. 2 demonstrates significantly lower HRR values in rheumatoid arthritis patients compared to non-rheumatoid arthritis individuals, with the mean difference between groups reaching statistical significance (*P* < 0.0001). This finding further supports the potential protective association between higher HRR levels and reduced rheumatoid arthritis risk.

**Table 1:** Baseline characteristics of adults in the United States stratified by quartiles of the hemoglobin-to-red blood cell distribution width ratio, using data from the National Health and Nutrition Examination Survey, 2009–2018.

Variables	Total (*n* = 22,352)	HRR				P-value
		Quartile 1 (*n* = 5589)	Quartile 2 (*n* = 5619)	Quartile 3 (*n* = 5602)	Quartile 4 (*n* = 5542)	
Age, n (%), years						<0.001
20–40	8203 (36.70)	1615 (28.90)	1785 (31.77)	2167 (38.68)	2636 (47.56)	
40–65	9684 (43.32)	2317 (41.46)	2558 (45.52)	2495 (44.54)	2314 (41.75)	
≥65	4465 (19.98)	1657 (29.65)	1276 (22.71)	940 (16.78)	592 (10.68)	
Gender, n (%)						<0.001
Male	11,309 (50.60)	1360 (24.33)	2002 (35.63)	3249 (58.00)	4698 (84.77)	
Female	11,043 (49.40)	4229 (75.67)	3617 (64.37)	2353 (42.00)	844 (15.23)	
Race, n (%)						<0.001
Mexican American	3517 (15.73)	746 (13.35)	856 (15.23)	917 (16.37)	998 (18.01)	
Other Hispanic	2413 (10.80)	587 (10.50)	666 (11.85)	637 (11.37)	523 (9.44)	
Non-Hispanic White	8239 (36.86)	1385 (24.78)	1943 (34.58)	2281 (40.72)	2630 (47.46)	
Non-Hispanic Black	4863 (21.76)	2133 (38.16)	1305 (23.22)	885 (15.80)	540 (9.74)	
Other Race	3320 (14.85)	738 (13.20)	849 (15.11)	882 (15.74)	851 (15.36)	
PIR, n (%)						<0.001
<1.3	7503 (33.57)	2105 (37.66)	1870 (33.28)	1769 (31.58)	1759 (31.74)	
1.3–3.5	8309 (37.17)	2183 (39.06)	2059 (36.64)	2001 (35.72)	2066 (37.28)	
≥3.5	6540 (29.26)	1301 (23.28)	1690 (30.08)	1832 (32.70)	1717 (30.98)	
Smoking status, n (%)						<0.001
Every day	7645 (34.20)	1752 (31.35)	1831 (32.59)	1960 (34.99)	2102 (37.93)	
Some days	2167 (9.69)	454 (8.12)	529 (9.41)	573 (10.23)	611 (11.02)	
Not at all	12,540 (56.10)	3383 (60.53)	3259 (58.00)	3069 (54.78)	2829 (51.05)	
Past-year alcohol drinking, n (%)						<0.001
Non-drinker	7601 (34.01)	2229 (39.88)	2123 (37.78)	1785 (31.86)	1464 (26.42)	
1–3 drinks	9302 (41.62)	2260 (40.44)	2345 (41.73)	2411 (43.04)	2286 (41.25)	
≥4 drinks	5449 (24.38)	1100 (19.68)	1151 (20.48)	1406 (25.10)	1792 (32.33)	
BMI, n (%)						<0.001
Underweight	373 (1.67)	92 (1.65)	103 (1.83)	107 (1.91)	71 (1.28)	
Normal weight	6314 (28.25)	1436 (25.69)	1627 (28.96)	1688 (30.13)	1563 (28.20)	
Overweight	7373 (32.99)	1552 (27.77)	1806 (32.14)	1906 (34.02)	2109 (38.05)	
Obese	8292 (37.10)	2509 (44.89)	2083 (37.07)	1901 (33.93)	1799 (32.46)	
Diabetes mellitus, n (%)						<0.001
Yes	2740 (12.26)	1050 (18.79)	718 (12.78)	548 (9.78)	424 (7.65)	
No	19,112 (85.50)	4380 (78.37)	4753 (84.59)	4959 (88.52)	5020 (90.58)	
Borderline	500 (2.24)	159 (2.84)	148 (2.63)	95 (1.70)	98 (1.77)	
Hypertension, n (%)						<0.001
Yes	7363 (32.94)	2445 (43.75)	1919 (34.15)	1633 (29.15)	1366 (24.65)	
No	14,989 (67.06)	3144 (56.25)	3700 (65.85)	3969 (70.85)	4176 (75.35)	
Education level, n (%)						<0.001
Less than 12th grade	5356 (23.96)	1458 (26.09)	1345 (23.94)	1296 (23.13)	1257 (22.68)	
High school	4998 (22.36)	1283 (22.96)	1218 (21.68)	1248 (22.28)	1249 (22.54)	
College or more	11,998 (53.68)	2848 (50.96)	3056 (54.39)	3058 (54.59)	3036 (54.78)	
Rheumatoid arthritis, n (%)						<0.001
Yes	1336 (5.98)	525 (9.39)	369 (6.57)	256 (4.57)	186 (3.36)	
No	21,016 (94.02)	5064 (90.61)	5250 (93.43)	5346 (95.43)	5356 (96.64)	
Hemoglobin (g/dL, Mean ± SD)	14.02 ± 1.54	12.27 ± 1.23	13.66 ± 0.75	14.51 ± 0.77	15.65 ± 0.88	<0.001
RDW (%, Mean ± SD)	13.45 ± 1.40	14.90 ± 1.86	13.35 ± 0.70	12.98 ± 0.65	12.56 ± 0.61	<0.001
HRR	1.06 ± 0.17	0.84 ± 0.13	1.02 ± 0.03	1.12 ± 0.03	1.25 ± 0.06	<0.001

Mean ± SD for continuous variables: the P value was calculated by the weighted linear regression model; (%) for categorical variables: the P value was calculated by the weighted chi-square test. Abbreviation: PIR, family poverty income ratio; BMI: body mass index; RDW, red blood cell distribution width; HRR, hemoglobin-to-red blood cell distribution width ratio.

### 3.2 The relationship between HRR and rheumatoid arthritis

Table 2 presents the results of the multivariable logistic regression model examining the association between HRR and rheumatoid arthritis. In the unadjusted model, each unit increase in HRR was associated with an 88 % reduction in the risk of rheumatoid arthritis (OR = 0.12, 95 % CI: 0.09,0.16). After adjusting for age, gender, race, PIR, educational level, metabolic disorders (diabetes and hypertension), and lifestyle factors (smoking, alcohol consumption, and BMI), HRR remained independently associated with a 56 % reduction in rheumatoid arthritis risk (OR = 0.44, 95 % CI: 0.33,0.59). When HRR was analyzed as a categorical variable (quartiles), Model 1 demonstrated a progressive decrease in rheumatoid arthritis risk across increasing HRR quartiles (quartile 2: OR = 0.68; quartile 3: OR = 0.46; quartile 4: OR = 0.33). Participants in the highest quartile (95 % CI: 0.28,0.40) exhibited a 67 % lower risk of rheumatoid arthritis compared to those in the lowest quartile. In the fully adjusted Model 2, the associations were somewhat attenuated but remained statistically significant (quartile 2: OR = 0.85; quartile 3: OR = 0.73; quartile 4: OR = 0.68), with individuals in quartile 4 (95 % CI: 0.56,0.83) showing a 32 % reduced risk of rheumatoid arthritis compared to quartile 1.

**Table 2:** Multivariable logistic regression analysis of the association between hemoglobin-to-red blood cell distribution width ratio and rheumatoid arthritis among adults in the United States, using data from the National Health and Nutrition Examination Survey, 2009–2018.

	Model 1	Model 2
	OR (95 %CI)	OR (95 %CI)
HRR	0.12 (0.09,0.16)	0.44 (0.30,0.64)
Quartile 1	1.00	1.00
Quartile 2	0.68 (0.59,0.78)	0.85 (0.73,0.98)
Quartile 3	0.46 (0.40,0.54)	0.73 (0.62,0.86)
Quartile 4	0.33 (0.28,0.40)	0.68 (0.56,0.83)
P for trend	<0.001	<0.001

Model 1: No covariates were adjusted; Model 2: Adjust for age, gender, race, Education level, PIR, Smoking status, Past-year alcohol drinking, BMI, Diabetes mellitus and Hypertension.

Abbreviation: OR: odds ratio; CI: confidence interval; PIR, family poverty income ratio; BMI: body mass index; HRR, hemoglobin-to-red blood cell distribution width ratio.

**Fig. 3:**
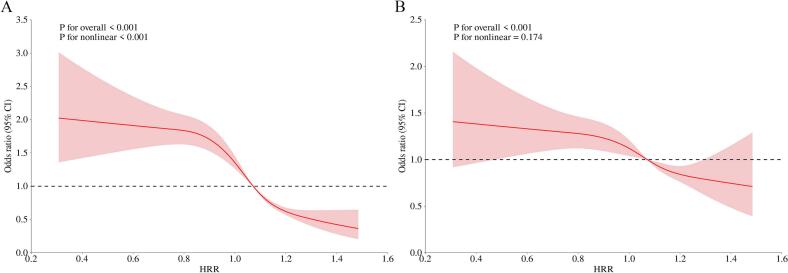
Restricted cubic spline plots illustrating the relationship between the hemoglobin-to-red blood cell distribution width ratio and the odds of rheumatoid arthritis among adults in the United States, based on data from the National Health and Nutrition Examination Survey, 2009–2018. The solid line and shadow represented the odds ratio of rheumatoid arthritis and 95 % confidence interval, respectively. A: no covariates were adjusted. B: all covariates were adjusted. Abbreviation: HRR, hemoglobin-to-red blood cell distribution width ratio. (For interpretation of the references to colour in this figure legend, the reader is referred to the web version of this article.)

Fig. 3 displays the notable correlations between HRR and rheumatoid arthritis risk, which were detected using RCS. In the unadjusted model (Fig. 3A), a pronounced inverse correlation was observed between HRR levels and rheumatoid arthritis risk (P for overall <0.001), with a statistically significant nonlinear component (P for nonlinear <0.001). Following comprehensive adjustment for all covariates (Fig. 3B), the inverse association remained statistically significant (P for overall <0.001), while the nonlinear relationship was no longer evident (P for nonlinear = 0.174). The adjusted analysis demonstrated an approximately linear inverse correlation, characterized by narrower confidence intervals, suggesting enhanced robustness of the association after multivariable adjustment.

## 4 Discussion

This study represents the first investigation of the association between HRR and rheumatoid arthritis risk using nationally representative data from the 2009–2018 NHANES cycles. Our findings reveal a significant negative correlation between HRR measurements and rheumatoid arthritis susceptibility. In multivariable logistic regression analyses, HRR as a continuous variable showed a robust unadjusted association with rheumatoid arthritis risk, with each unit increase in HRR associated with an 88 % risk reduction (Model 1). This strong crude association suggests potential confounding effects. Following full adjustment for covariates, HRR maintained an independent protective effect, with each unit increment associated with a 56 % risk reduction (Model 2), indicating partial mediation through the adjusted covariates. When analyzed as a categorical variable, higher HRR levels demonstrated progressively lower rheumatoid arthritis risk in the unadjusted model (Model 1), with the highest quartile showing a 67 % risk reduction compared to quartile 1. This protective effect, though attenuated to 32 % in the fully adjusted model (Model 2), remained clinically significant, supporting HRR's utility as a stratification marker. In the univariate restricted cubic spline analysis, a significant inverse correlation was observed between HRR levels and rheumatoid arthritis risk, characterized by a nonlinear relationship (P for nonlinear <0.001). Following comprehensive adjustment for covariates in the multivariable RCS model, the inverse association remained statistically significant (P for overall <0.001), while the nonlinear component was no longer evident (P for nonlinear = 0.174). This transition suggests that the initial nonlinear pattern was partially attributable to confounding factors, with the independent effect of HRR better represented by a linear model, where each unit increment in HRR consistently correlates with reduced rheumatoid arthritis risk. The synthesis of findings from the multivariable logistic regression models and RCS analyses consistently reveals a significant negative correlation between HRR measurements and rheumatoid arthritis susceptibility.

Anemia of chronic disease represents a prevalent extra-articular manifestation of rheumatoid arthritis, affecting up to 50 % of patients (Papadaki et al., 2002). While the precise pathogenesis of ACD remains incompletely understood, current evidence suggests a multifactorial mechanism driven by proinflammatory cytokines (Weiss et al., 2019). Notably, interleukin-6 induces hepatic overexpression of hepcidin, a key regulator of iron homeostasis, thereby impairing intestinal iron absorption and sequestering iron within macrophages (Camaschella et al., 2020). This dysregulation culminates in functional iron deficiency, a hallmark of ACD (Weiss and Goodnough, 2005). Simultaneously, tumor necrosis factor-alpha suppresses erythroid progenitor differentiation in the bone marrow and diminishes erythropoietin responsiveness, further exacerbating anemia (De Simone et al., 2014). Elevated reactive oxygen species levels in rheumatoid arthritis patients contribute to erythrocyte membrane damage through lipid peroxidation, enhancing red blood cell fragility and shortening their lifespan (Smith et al., 1992). These pathophysiological alterations are clinically reflected in elevated red cell distribution width and heightened oxidative stress (Oustamanolakis et al., 2011). The resultant anemia aggravates tissue hypoxia, activating hypoxia-inducible factor-1α (HIF-1α) (Chen et al., 2023). This transcription factor amplifies IL-6 and vascular endothelial growth factor expression, creating a proinflammatory feedback loop that perpetuates synovitis and promotes pannus formation (Sato and Takeda, 2023). Emerging research highlights IL-6-mediated induction of peptidylarginine deiminase (PAD) in synovial cells (Pérez-Pérez et al., 2024). PAD enzymes catalyze the citrullination of self-proteins, generating neoepitopes that trigger anti-citrullinated protein antibody production, while TNF-α enhances Th17 cell differentiation through nuclear factor kappa-B signaling and impairs regulatory T cell function, collectively driving rheumatoid arthritis-associated autoimmunity and joint destruction (Jie et al., 2023). Chronic hypoxic conditions secondary to reduced hemoglobin levels activate HIF-1α, facilitating the transition of fibroblast-like synoviocytes to an invasive phenotype characterized by matrix metalloproteinase-9 secretion, which accelerates cartilage degradation (Chellini et al., 2023). Therefore, a diminished HRR may not only serve as a biomarker for advancing ACD but also portend systemic vascular endothelial injury and an elevated risk of cardiovascular events, positioning it as a composite indicator for integrated multisystem management in rheumatoid arthritis.

Our study presents several notable advantages. Primarily, the analysis is the first to reveal a significant negative correlation between HRR measurements and rheumatoid arthritis susceptibility. Additionally, it benefits from an extensive dataset sourced from the NHANES repository. Furthermore, this study utilized a combination of multivariable logistic regression and restricted cubic spline analysis to systematically evaluate the association between HRR and rheumatoid arthritis, yielding robust results even after adjustment for multiple covariates. Several limitations should be acknowledged in this investigation. This study was based on a cross-sectional design, which precludes the ability to draw causal inferences between HRR and rheumatoid arthritis. Second, the effects of rheumatoid arthritis treatment drugs on hemoglobin and RDW were not adjusted, which may underestimate the independent effect of HRR. Third, the use of anti-inflammatory or immunosuppressive medications by rheumatoid arthritis patients may influence hematological parameters, potentially introducing bias into the estimated association between HRR and rheumatoid arthritis. Future studies are recommended to collect detailed medication data to assess and adjust for this potential confounding effect. Additionally, the absence of other direct inflammatory markers and iron metabolism indicators limits the depth of mechanistic exploration.

## 5 Conclusions

Our findings reveal a significant negative correlation between HRR measurements and rheumatoid arthritis susceptibility, suggesting that individuals with higher HRR levels may have a lower risk of developing rheumatoid arthritis. This discovery underscores the potential utility of HRR assessment in identifying individuals susceptible to rheumatoid arthritis, emphasizing its diagnostic significance. Nevertheless, additional investigations are required to corroborate these observations.

## CRediT authorship contribution statement

**Nian Kuang:** Writing – review & editing, Writing – original draft, Visualization, Validation, Supervision, Software, Resources, Project administration, Methodology, Investigation, Formal analysis, Data curation, Conceptualization. **Jing Liu:** Writing – review & editing, Writing – original draft, Formal analysis. **Zhaoduan Hu:** Writing – review & editing, Writing – original draft, Software, Formal analysis. **Yanxia Wu:** Writing – review & editing, Writing – original draft, Validation, Formal analysis. **Rui Peng:** Writing – review & editing, Writing – original draft, Validation, Supervision, Software, Resources, Methodology, Investigation, Formal analysis, Conceptualization.

## Consent for publication

Not applicable.

## Ethics statement

The study protocol (Protocol Number: Protocol #2009–2018) was approved by the National Center for Health Statistics (NCHS) Research Ethics Review Board (ERB) (https://www.cdc.gov/nchs/nhanes/about/erb.html?CDC_AAref_Val=https://www.cdc.gov/nchs/nhanes/irba98.htm).

## Submission declaration and verification

We confirm that the manuscript contains novel research findings that have not been previously published and are not presently under consideration elsewhere. All authors have thoroughly reviewed and agreed to submit this manuscript.

## Funding

This study received no funding from public, commercial, or non-profit sectors.

## Declaration of competing interest

The authors declare that they have no known competing financial interests or personal relationships that could have appeared to influence the work reported in this paper.

## Data Availability

The datasets used for these analyses are publicly available (https://www.cdc.gov/nchs/nhanes/index.htm). All necessary permissions for data use have been obtained.
